# Precision Phenotyping of Mild Behavioral Impairment and the Early Detection of Dementia

**DOI:** 10.1002/alz.094252

**Published:** 2025-01-09

**Authors:** Ipsit V Vahia

**Affiliations:** ^1^ Harvard Medical School, Belmont, MA USA

## Abstract

There is growing recognition of the importance of changes in behavior as an early clinical marker of the onset of Alzheimer’s disease. Behavior symptoms may precede the onset of cognitive symptoms by as many as three years. However, these symptoms are often confused for primary psychiatric pathology and there is an urgent need for markers that can help quantify behaviors with the eventual goal of helping distinguish behavior changes related to psychiatric pathology from behavior changes related to the onset of dementia. This talk will summarize the state of knowledge on mild behavioral impairment, and present data from three studies demonstrating how a range of sensor‐derived biomarkers can quantify subtle changes in behavior with precision. The talk will include case presentations around detection of apathy, agitation, pacing. It will also demonstrate how these markers can identify response to medications, which may be one way of distinguishing the presence of psychiatric versus neurodegenerative pathology. Finally, the talk will present data from ongoing clinical trials around how these biomarkers may translate into research and eventually practice.

